# 3-[2-Cyclo­propyl-1-(2-fluoro­phen­yl)-2-oxoeth­yl]-5-(4-methyl­sulfanyl­benzyl­idene)-1,3-thia­zolidine-2,4-dione

**DOI:** 10.1107/S1600536812051987

**Published:** 2013-01-04

**Authors:** J. Suresh, M. Venkateshan, S. Ponnuchamy, R. Ranjith Kumar, P. L. Nilantha Lakshman

**Affiliations:** a Department of Physics, The Madura College, Madurai 625 011, India; b Orchid Chemicals & Pharmaceuticals Ltd, R&D Center, Chennai 600 119, India; c Department of Organic Chemistry, School of Chemistry, Madurai Kamaraj University, Madurai 625 021, India; d Department of Food Science and Technology, University of Ruhuna, Mapalana, Kamburupitiya 81100, Sri Lanka

## Abstract

In the title compound, C_22_H_18_FNO_3_S_2_, the five-membered thia­zolidine ring is planar (r.m.s. deviation = 0.003 Å) and forms dihedral angles of 70.2 (3), 73.16 (17) and 10.32 (14)° with the cyclo­propane, fluoro­benzene and methyl­thio­benzene rings, respectively. The sum of the bond angles around the thia­zolidine ring N atom (359.6°) indicates *sp* ^2^ hybridization. The mol­ecular structure features intra­molecular C—H⋯S, C—H⋯F and C—H⋯O inter­actions. In the crystal, no significant inter­molecular contacts were apparent.

## Related literature

For general properties of thia­zolidines, see: Botti *et al.* (1996 ▶); Spiegelman (1998 ▶); Day (1999 ▶); Barreca *et al.* (2002 ▶).
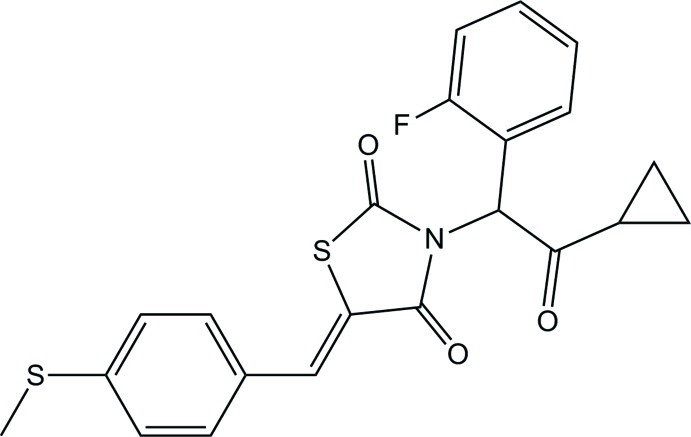


## Experimental

### 

#### Crystal data

C_22_H_18_FNO_3_S_2_
*M* *_r_* = 427.49
Monoclinic, 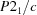

*a* = 7.657 (3) Å
*b* = 15.799 (5) Å
*c* = 17.425 (6) Å
β = 95.641 (5)°
*V* = 2097.7 (13) Å^3^
*Z* = 4
Mo *K*α radiation
μ = 0.29 mm^−1^
*T* = 293 K
0.23 × 0.21 × 0.19 mm

#### Data collection

Bruker Kappa APEXII diffractometer
Absorption correction: multi-scan (*SADABS*; Sheldrick, 1996 ▶) *T*_min_ = 0.967, *T*_max_ = 0.974
19651 measured reflections
3838 independent reflections
2429 reflections with *I* > 2σ(*I*)
*R*_int_ = 0.033

#### Refinement

*R*[*F* ^2^ > 2σ(*F* ^2^)] = 0.047
*wR*(*F* ^2^) = 0.149
*S* = 1.02
3838 reflections
262 parameters
H-atom parameters constrained
Δρ_max_ = 0.32 e Å^−3^
Δρ_min_ = −0.26 e Å^−3^

### 

Data collection: *APEX2* (Bruker, 2004 ▶); cell refinement: *SAINT* (Bruker, 2004 ▶); data reduction: *SAINT*; program(s) used to solve structure: *SHELXS97* (Sheldrick, 2008 ▶); program(s) used to refine structure: *SHELXL97* (Sheldrick, 2008 ▶); molecular graphics: *PLATON* (Spek, 2009 ▶); software used to prepare material for publication: *SHELXL97*.

## Supplementary Material

Crystal structure: contains datablock(s) global, I. DOI: 10.1107/S1600536812051987/tk5185sup1.cif

Structure factors: contains datablock(s) I. DOI: 10.1107/S1600536812051987/tk5185Isup2.hkl

Supplementary material file. DOI: 10.1107/S1600536812051987/tk5185Isup3.cml

Additional supplementary materials:  crystallographic information; 3D view; checkCIF report

## Figures and Tables

**Table 1:** Hydrogen-bond geometry (Å, °)

*D*—H⋯*A*	*D*—H	H⋯*A*	*D*⋯*A*	*D*—H⋯*A*
C4—H4⋯F	0.98	2.35	2.740 (4)	103
C4—H4⋯O2	0.98	2.33	2.792 (4)	108
C15—H15⋯O2	0.93	2.53	2.884 (4)	103
C17—H17⋯S1	0.93	2.55	3.238 (3)	131

**Fig. 1.**
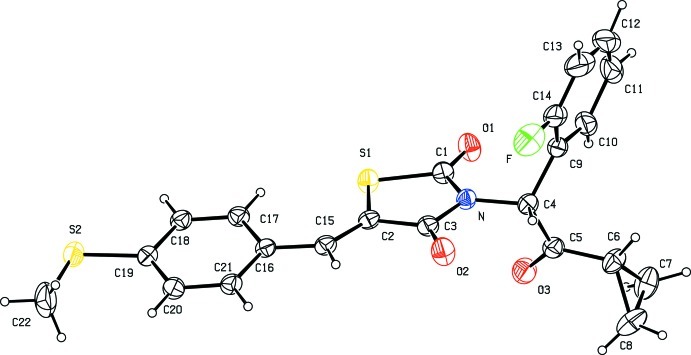
The molecular structure of the title compound, showing 30% probability displacement ellipsoids and the atom-numbering scheme.

## supplementary crystallographic information

### Comment

Thiazolidines are an important class of heteroaromatic compounds and have widespread applications from ranging from pharmaceuticals (Barreca *et al.*, 2002) to materials (Botti *et al.*, 1996). Thiazolidinediones (TZDs), which are known to sensitize tissues to insulin, have been developed and clinically used as anti-diabetic agents. They have been shown to reduce plasma glucose, lipid, and insulin levels, and are used for the treatment of type 2 diabetes (Day, 1999; Spiegelman, 1998). In view of this we have synthesized the title compound to study its crystal structure.

In the title compound, Fig. 1, the five-membered thiazolidine is nearly planar with a r.m.s. deviation of 0.003 Å. The 4-methyl sulfonyl benzylidine ring is nearly coplanar with the central thiazolidine ring as indicated by the dihedral angle of 8.35 (1)°. The 2-fluorobenzene and cyclopropyl rings make dihedral angles of 73.16 (17) and 70.2 (3)° with the thiazolidine ring, respectively. The sum of bond angles around N is 359.6° which confirms *sp*^2 ^hybridization. In the cyclopropyl ring the mean C—C bond length is 1.504 (3) Å and the mean C—C—C bond angle is 60.0 (2)°; these are unexceptional. The molecular structure features intramolecular C—H···S, C—H···F C—H···O interactions (Table 1).

### Experimental

A mixture of 5-(4-methylsulfanyl-benzylidenethiazolidine-2,4-dione (1 mmol), 2-bromo-1-cyclopropyl-2-(2-fluorophenyl)ethanone (1 mmol) and sodium bicarbonate (3 mmol) were taken in DMF and stirred for 6 h at 25–35 °C. After completion of the reaction as evident by TLC, (eluent: 7:3 / hexane:ethyl acetate) the mixture was poured into water and extracted with ethyl acetate. The organic layer was washed with brine (20 ml) twice and then with water. The solvent was distilled off to obtain a viscous paste to which diisopropyl ether (5 volumes) was added. The resultant solid was filtered and washed with diisopropyl ether. The product was recrystallized from its methanol solution. Melting point: 414.5–415.9 Yield: 83.5%

### Refinement

H atoms were placed at calculated positions and allowed to ride on their carrier atoms with C—H = 0.93–0.98 Å. *U*_iso_ = 1.2*U*_eq_(C) for CH_2_ and CH groups and *U*_iso_ = 1.5*U*_eq_(C) for the CH_3_ group. The (0 1 1) reflection was probably affected by the beam-stop and was omitted from the refinement.

### Crystal data

**Table:**

C_22_H_18_FNO_3_S_2_	*F*(000) = 888
*M**_r_* = 427.49	*D*_x_ = 1.354 Mg m^−^^3^
Monoclinic, *P*2_1_/*c*	Mo *K*α radiation, λ = 0.71073 Å
Hall symbol: -P 2ybc	Cell parameters from 2000 reflections
*a* = 7.657 (3) Å	θ = 2–25°
*b* = 15.799 (5) Å	µ = 0.29 mm^−^^1^
*c* = 17.425 (6) Å	*T* = 293 K
β = 95.641 (5)°	Block, colourless
*V* = 2097.7 (13) Å^3^	0.23 × 0.21 × 0.19 mm
*Z* = 4	

### Data collection

**Table:**

Bruker Kappa APEXII diffractometer	3838 independent reflections
Radiation source: fine-focus sealed tube	2429 reflections with *I* > 2σ(*I*)
Graphite monochromator	*R*_int_ = 0.033
Detector resolution: 0 pixels mm^-1^	θ_max_ = 25.4°, θ_min_ = 2.4°
ω and φ scans	*h* = −5→9
Absorption correction: multi-scan (*SADABS*; Sheldrick, 1996)	*k* = −18→19
*T*_min_ = 0.967, *T*_max_ = 0.974	*l* = −21→20
19651 measured reflections	

### Refinement

**Table:**

Refinement on *F*^2^	Primary atom site location: structure-invariant direct methods
Least-squares matrix: full	Secondary atom site location: difference Fourier map
*R*[*F*^2^ > 2σ(*F*^2^)] = 0.047	Hydrogen site location: inferred from neighbouring sites
*wR*(*F*^2^) = 0.149	H-atom parameters constrained
*S* = 1.02	*w* = 1/[σ^2^(*F*_o_^2^) + (0.0683*P*)^2^ + 0.8976*P*] where *P* = (*F*_o_^2^ + 2*F*_c_^2^)/3
3838 reflections	(Δ/σ)_max_ < 0.001
262 parameters	Δρ_max_ = 0.32 e Å^−^^3^
0 restraints	Δρ_min_ = −0.26 e Å^−^^3^

### Special details

**Table:**

Geometry. All e.s.d.'s (except the e.s.d. in the dihedral angle between two l.s. planes) are estimated using the full covariance matrix. The cell e.s.d.'s are taken into account individually in the estimation of e.s.d.'s in distances, angles and torsion angles; correlations between e.s.d.'s in cell parameters are only used when they are defined by crystal symmetry. An approximate (isotropic) treatment of cell e.s.d.'s is used for estimating e.s.d.'s involving l.s. planes.
Refinement. Refinement of *F*^2^ against ALL reflections. The weighted *R*-factor *wR* and goodness of fit *S* are based on *F*^2^, conventional *R*-factors *R* are based on *F*, with *F* set to zero for negative *F*^2^. The threshold expression of *F*^2^ > σ(*F*^2^) is used only for calculating *R*-factors(gt) *etc*. and is not relevant to the choice of reflections for refinement. *R*-factors based on *F*^2^ are statistically about twice as large as those based on *F*, and *R*- factors based on ALL data will be even larger.

### Fractional atomic coordinates and isotropic or equivalent isotropic displacement parameters (Å^2^)

**Table:**

	*x*	*y*	*z*	*U*_iso_*/*U*_eq_	
C1	−0.0768 (4)	0.07851 (19)	0.35525 (16)	0.0603 (7)	
C2	0.2318 (3)	0.02818 (18)	0.34213 (15)	0.0539 (7)	
C3	0.1614 (4)	0.08537 (18)	0.28013 (16)	0.0589 (7)	
C4	−0.1107 (4)	0.16162 (18)	0.23261 (15)	0.0575 (7)	
H4	−0.0273	0.1811	0.1972	0.069*	
C5	−0.2414 (4)	0.1066 (2)	0.18456 (17)	0.0648 (8)	
C6	−0.3427 (4)	0.1473 (2)	0.11919 (18)	0.0769 (9)	
H6	−0.3314	0.2089	0.1151	0.092*	
C7	−0.5155 (5)	0.1103 (3)	0.0911 (2)	0.1003 (13)	
H7A	−0.5547	0.0611	0.1181	0.120*	
H7B	−0.6082	0.1489	0.0719	0.120*	
C8	−0.3702 (6)	0.0993 (3)	0.0454 (2)	0.1102 (14)	
H8A	−0.3720	0.1311	−0.0023	0.132*	
H8B	−0.3185	0.0433	0.0439	0.132*	
C9	−0.1842 (4)	0.24062 (19)	0.26535 (16)	0.0640 (8)	
C10	−0.3536 (5)	0.2472 (2)	0.28457 (19)	0.0892 (11)	
H10	−0.4307	0.2019	0.2766	0.107*	
C11	−0.4098 (8)	0.3236 (4)	0.3166 (2)	0.1203 (18)	
H11	−0.5241	0.3296	0.3295	0.144*	
C12	−0.2909 (11)	0.3889 (3)	0.3283 (3)	0.137 (3)	
H12	−0.3257	0.4385	0.3511	0.164*	
C13	−0.1253 (10)	0.3835 (3)	0.3079 (3)	0.131 (2)	
H13	−0.0495	0.4295	0.3144	0.157*	
C14	−0.0726 (6)	0.3109 (2)	0.27810 (19)	0.0832 (10)	
C15	0.3898 (3)	−0.00777 (18)	0.34052 (16)	0.0566 (7)	
H15	0.4507	0.0093	0.2996	0.068*	
C16	0.4815 (3)	−0.06842 (17)	0.39142 (15)	0.0523 (6)	
C17	0.4287 (4)	−0.09486 (18)	0.46215 (16)	0.0589 (7)	
H17	0.3247	−0.0741	0.4782	0.071*	
C18	0.5269 (4)	−0.15058 (19)	0.50819 (16)	0.0605 (7)	
H18	0.4890	−0.1671	0.5550	0.073*	
C19	0.6831 (4)	−0.18290 (19)	0.48574 (16)	0.0611 (7)	
C20	0.7347 (4)	−0.1587 (2)	0.41522 (17)	0.0677 (8)	
H20	0.8375	−0.1804	0.3987	0.081*	
C21	0.6358 (3)	−0.10303 (19)	0.36966 (16)	0.0609 (7)	
H21	0.6730	−0.0877	0.3224	0.073*	
C22	0.9974 (6)	−0.2703 (4)	0.5069 (3)	0.142 (2)	
H22A	1.0729	−0.3067	0.5394	0.212*	
H22B	1.0571	−0.2180	0.4990	0.212*	
H22C	0.9670	−0.2974	0.4581	0.212*	
N	−0.0076 (3)	0.11071 (14)	0.29086 (12)	0.0543 (6)	
O1	−0.2202 (3)	0.09354 (14)	0.37424 (13)	0.0807 (7)	
O2	0.2345 (3)	0.10769 (15)	0.22512 (13)	0.0868 (7)	
O3	−0.2558 (4)	0.03221 (15)	0.19915 (15)	0.0952 (8)	
F	0.0895 (4)	0.30462 (16)	0.25692 (14)	0.1184 (8)	
S1	0.07690 (9)	0.01156 (5)	0.40749 (4)	0.0652 (3)	
S2	0.80371 (12)	−0.24929 (6)	0.55177 (5)	0.0834 (3)	

### Atomic displacement parameters (Å^2^)

**Table:**

	*U*^11^	*U*^22^	*U*^33^	*U*^12^	*U*^13^	*U*^23^
C1	0.0589 (16)	0.0646 (18)	0.0587 (18)	0.0013 (14)	0.0130 (14)	0.0060 (15)
C2	0.0573 (15)	0.0623 (17)	0.0433 (15)	−0.0036 (13)	0.0116 (12)	−0.0021 (13)
C3	0.0620 (16)	0.0637 (18)	0.0523 (17)	0.0018 (14)	0.0132 (14)	0.0010 (14)
C4	0.0649 (16)	0.0608 (18)	0.0476 (16)	0.0013 (14)	0.0102 (13)	0.0069 (14)
C5	0.0770 (19)	0.062 (2)	0.0559 (18)	0.0030 (16)	0.0072 (15)	0.0013 (15)
C6	0.094 (2)	0.079 (2)	0.0565 (19)	−0.0150 (18)	−0.0036 (17)	0.0109 (17)
C7	0.086 (2)	0.123 (3)	0.089 (3)	−0.018 (2)	−0.004 (2)	0.033 (2)
C8	0.122 (3)	0.153 (4)	0.054 (2)	−0.023 (3)	0.002 (2)	−0.007 (2)
C9	0.091 (2)	0.0552 (19)	0.0455 (16)	0.0119 (17)	0.0063 (15)	0.0055 (14)
C10	0.113 (3)	0.092 (3)	0.065 (2)	0.044 (2)	0.022 (2)	0.0144 (19)
C11	0.163 (4)	0.132 (4)	0.071 (3)	0.078 (4)	0.034 (3)	0.033 (3)
C12	0.263 (8)	0.078 (3)	0.070 (3)	0.063 (5)	0.022 (4)	0.011 (3)
C13	0.257 (7)	0.064 (3)	0.068 (3)	−0.001 (4)	−0.005 (4)	0.008 (2)
C14	0.134 (3)	0.063 (2)	0.0523 (19)	0.007 (2)	0.007 (2)	0.0114 (17)
C15	0.0565 (15)	0.0679 (18)	0.0470 (16)	−0.0024 (14)	0.0129 (12)	−0.0016 (14)
C16	0.0510 (14)	0.0604 (17)	0.0456 (15)	−0.0017 (13)	0.0055 (12)	−0.0055 (13)
C17	0.0545 (15)	0.0688 (19)	0.0547 (17)	0.0030 (14)	0.0121 (13)	−0.0046 (14)
C18	0.0659 (17)	0.0689 (19)	0.0475 (16)	0.0000 (15)	0.0087 (14)	−0.0011 (14)
C19	0.0616 (16)	0.0678 (19)	0.0524 (18)	0.0053 (14)	−0.0018 (14)	−0.0051 (14)
C20	0.0572 (16)	0.088 (2)	0.0592 (18)	0.0125 (16)	0.0126 (14)	−0.0009 (17)
C21	0.0596 (16)	0.077 (2)	0.0477 (16)	0.0033 (15)	0.0133 (13)	0.0007 (15)
C22	0.106 (3)	0.218 (6)	0.103 (3)	0.088 (3)	0.020 (3)	0.042 (3)
N	0.0560 (12)	0.0618 (14)	0.0459 (13)	0.0031 (11)	0.0089 (10)	0.0055 (11)
O1	0.0627 (12)	0.0963 (17)	0.0874 (16)	0.0178 (11)	0.0300 (11)	0.0260 (13)
O2	0.0850 (14)	0.1093 (18)	0.0723 (14)	0.0220 (13)	0.0382 (12)	0.0331 (13)
O3	0.127 (2)	0.0603 (15)	0.0921 (18)	−0.0026 (14)	−0.0193 (15)	0.0020 (13)
F	0.134 (2)	0.1123 (19)	0.1068 (18)	−0.0420 (16)	0.0020 (15)	0.0186 (14)
S1	0.0581 (4)	0.0814 (6)	0.0582 (5)	0.0072 (4)	0.0169 (3)	0.0164 (4)
S2	0.0848 (6)	0.1018 (7)	0.0621 (5)	0.0229 (5)	−0.0005 (4)	0.0087 (5)

### Geometric parameters (Å, º)

**Table:**

C1—O1	1.201 (3)	C10—H10	0.9300
C1—N	1.384 (3)	C11—C12	1.378 (8)
C1—S1	1.767 (3)	C11—H11	0.9300
C2—C15	1.340 (4)	C12—C13	1.353 (8)
C2—C3	1.470 (4)	C12—H12	0.9300
C2—S1	1.743 (3)	C13—C14	1.337 (6)
C3—O2	1.209 (3)	C13—H13	0.9300
C3—N	1.385 (3)	C14—F	1.333 (4)
C4—N	1.464 (3)	C15—C16	1.441 (4)
C4—C9	1.504 (4)	C15—H15	0.9300
C4—C5	1.515 (4)	C16—C21	1.388 (4)
C4—H4	0.9800	C16—C17	1.398 (4)
C5—O3	1.209 (4)	C17—C18	1.366 (4)
C5—C6	1.463 (4)	C17—H17	0.9300
C6—C7	1.486 (5)	C18—C19	1.391 (4)
C6—C8	1.490 (5)	C18—H18	0.9300
C6—H6	0.9800	C19—C20	1.381 (4)
C7—C8	1.441 (5)	C19—S2	1.752 (3)
C7—H7A	0.9700	C20—C21	1.363 (4)
C7—H7B	0.9700	C20—H20	0.9300
C8—H8A	0.9700	C21—H21	0.9300
C8—H8B	0.9700	C22—S2	1.774 (4)
C9—C10	1.375 (5)	C22—H22A	0.9600
C9—C14	1.406 (5)	C22—H22B	0.9600
C10—C11	1.413 (6)	C22—H22C	0.9600
			
O1—C1—N	125.7 (3)	C12—C11—H11	120.8
O1—C1—S1	123.8 (2)	C10—C11—H11	120.8
N—C1—S1	110.43 (19)	C13—C12—C11	122.5 (5)
C15—C2—C3	120.8 (2)	C13—C12—H12	118.8
C15—C2—S1	128.6 (2)	C11—C12—H12	118.8
C3—C2—S1	110.44 (19)	C14—C13—C12	118.9 (6)
O2—C3—N	122.3 (3)	C14—C13—H13	120.6
O2—C3—C2	126.7 (3)	C12—C13—H13	120.6
N—C3—C2	111.0 (2)	F—C14—C13	119.7 (5)
N—C4—C9	112.9 (2)	F—C14—C9	117.8 (3)
N—C4—C5	110.5 (2)	C13—C14—C9	122.5 (5)
C9—C4—C5	115.8 (2)	C2—C15—C16	131.0 (3)
N—C4—H4	105.6	C2—C15—H15	114.5
C9—C4—H4	105.6	C16—C15—H15	114.5
C5—C4—H4	105.6	C21—C16—C17	116.8 (3)
O3—C5—C6	122.6 (3)	C21—C16—C15	118.1 (2)
O3—C5—C4	120.7 (3)	C17—C16—C15	125.2 (2)
C6—C5—C4	116.6 (3)	C18—C17—C16	121.4 (3)
C5—C6—C7	118.0 (3)	C18—C17—H17	119.3
C5—C6—C8	117.8 (3)	C16—C17—H17	119.3
C7—C6—C8	57.9 (2)	C17—C18—C19	120.7 (3)
C5—C6—H6	116.7	C17—C18—H18	119.7
C7—C6—H6	116.7	C19—C18—H18	119.7
C8—C6—H6	116.7	C20—C19—C18	118.5 (3)
C8—C7—C6	61.2 (2)	C20—C19—S2	124.9 (2)
C8—C7—H7A	117.6	C18—C19—S2	116.6 (2)
C6—C7—H7A	117.6	C21—C20—C19	120.4 (3)
C8—C7—H7B	117.6	C21—C20—H20	119.8
C6—C7—H7B	117.6	C19—C20—H20	119.8
H7A—C7—H7B	114.8	C20—C21—C16	122.3 (3)
C7—C8—C6	60.9 (2)	C20—C21—H21	118.9
C7—C8—H8A	117.7	C16—C21—H21	118.9
C6—C8—H8A	117.7	S2—C22—H22A	109.5
C7—C8—H8B	117.7	S2—C22—H22B	109.5
C6—C8—H8B	117.7	H22A—C22—H22B	109.5
H8A—C8—H8B	114.8	S2—C22—H22C	109.5
C10—C9—C14	118.4 (3)	H22A—C22—H22C	109.5
C10—C9—C4	123.5 (3)	H22B—C22—H22C	109.5
C14—C9—C4	118.1 (3)	C1—N—C3	116.1 (2)
C9—C10—C11	119.5 (4)	C1—N—C4	122.7 (2)
C9—C10—H10	120.3	C3—N—C4	120.8 (2)
C11—C10—H10	120.3	C2—S1—C1	91.99 (13)
C12—C11—C10	118.4 (5)	C19—S2—C22	103.42 (18)
			
C15—C2—C3—O2	−2.4 (5)	C2—C15—C16—C21	171.7 (3)
S1—C2—C3—O2	−178.1 (3)	C2—C15—C16—C17	−9.2 (5)
C15—C2—C3—N	176.1 (2)	C21—C16—C17—C18	1.5 (4)
S1—C2—C3—N	0.4 (3)	C15—C16—C17—C18	−177.7 (3)
N—C4—C5—O3	3.6 (4)	C16—C17—C18—C19	−0.1 (4)
C9—C4—C5—O3	−126.4 (3)	C17—C18—C19—C20	−1.2 (4)
N—C4—C5—C6	−174.5 (2)	C17—C18—C19—S2	176.6 (2)
C9—C4—C5—C6	55.6 (4)	C18—C19—C20—C21	1.2 (5)
O3—C5—C6—C7	26.8 (5)	S2—C19—C20—C21	−176.4 (2)
C4—C5—C6—C7	−155.3 (3)	C19—C20—C21—C16	0.2 (5)
O3—C5—C6—C8	−39.7 (5)	C17—C16—C21—C20	−1.5 (4)
C4—C5—C6—C8	138.3 (3)	C15—C16—C21—C20	177.7 (3)
C5—C6—C7—C8	−106.9 (4)	O1—C1—N—C3	179.6 (3)
C5—C6—C8—C7	107.2 (4)	S1—C1—N—C3	0.0 (3)
N—C4—C9—C10	−98.6 (3)	O1—C1—N—C4	−6.4 (5)
C5—C4—C9—C10	30.1 (4)	S1—C1—N—C4	174.0 (2)
N—C4—C9—C14	80.3 (3)	O2—C3—N—C1	178.3 (3)
C5—C4—C9—C14	−151.0 (3)	C2—C3—N—C1	−0.3 (3)
C14—C9—C10—C11	−0.3 (5)	O2—C3—N—C4	4.2 (4)
C4—C9—C10—C11	178.6 (3)	C2—C3—N—C4	−174.3 (2)
C9—C10—C11—C12	−0.7 (6)	C9—C4—N—C1	58.9 (3)
C10—C11—C12—C13	2.3 (7)	C5—C4—N—C1	−72.5 (3)
C11—C12—C13—C14	−2.8 (8)	C9—C4—N—C3	−127.4 (3)
C12—C13—C14—F	179.3 (4)	C5—C4—N—C3	101.1 (3)
C12—C13—C14—C9	1.7 (6)	C15—C2—S1—C1	−175.6 (3)
C10—C9—C14—F	−177.9 (3)	C3—C2—S1—C1	−0.3 (2)
C4—C9—C14—F	3.1 (4)	O1—C1—S1—C2	−179.4 (3)
C10—C9—C14—C13	−0.2 (5)	N—C1—S1—C2	0.2 (2)
C4—C9—C14—C13	−179.2 (3)	C20—C19—S2—C22	2.2 (4)
C3—C2—C15—C16	−176.3 (3)	C18—C19—S2—C22	−175.6 (3)
S1—C2—C15—C16	−1.4 (5)		

### Hydrogen-bond geometry (Å, º)

**Table:**

*D*—H···*A*	*D*—H	H···*A*	*D*···*A*	*D*—H···*A*
C4—H4···F	0.98	2.35	2.740 (4)	103
C4—H4···O2	0.98	2.33	2.792 (4)	108
C15—H15···O2	0.93	2.53	2.884 (4)	103
C17—H17···S1	0.93	2.55	3.238 (3)	131
